# Aging: T cell metabolism within tumors

**DOI:** 10.18632/aging.100979

**Published:** 2016-06-08

**Authors:** Ying Zhang, Hildegund CJ Ertl

**Affiliations:** The Wistar Institute, Philadelphia, PA 19104, USA

**Keywords:** tumor infiltrating lymphocytes, metabolism, fatty acid beta-odidation, cancer immunotherapy

The incidence of cancer increases in the aged partially due to accumulations of genomic mutations, epigenetic dysregulations and the development of chronic inflammatory responses. In US, more than 50% of all cancer cases occur in patients over 65 [1]. Cancer immunotherapy, which has achieved significant breakthroughs in recent years, harnesses the patients' own immune system. However, the immune system deteriorates in the elderly, a phenomenon known as immunosenescence. Upon aging proliferative capacity and effector functions of CD4^+^ and CD8^+^ T cells decline, frequencies and functions of B cells and dendritic cells (DCs) decrease, while immunosuppressive cells (ISCs) increase, all of which dampen the efficacy of cancer immunotherapy [2]. We therefore need to develop novel treatment strategies that overcome defects of the aged immune system in order to improve the efficacy of cancer immunotherapy in elderly cancer patients.

The major goal of cancer immunotherapy is to elicit tumor antigen (TA)-specific effector T cells, which play a key role in controlling tumor growth. It is becoming increasingly clear that differentiation and effector functions of T cells are influenced by their metabolism. T cells at each stage of activation prefer to use distinct nutrient sources and metabolic pathways to support their biosynthesis and energy production [3]. TA-specific CD8^+^T cells, whether elicited by cancer vaccines or supplied by adoptive cell transfer, upon recognizing their cognate antigens within the tumor microenvironment (TME) will become highly activated and enhance glycolysis for biogenesis and ATP production. Glycolysis requires increased glucose uptake. This metabolic pathway is also preferred by tumor cells, a phenomenon known as the Warburg effect. Tumor cells consume glucose more efficiently than T cells. This in turn deprives tumor-infiltrating lymphocytes (TILs) of this key nutrient, which has been shown to dampen the cytolytic functions of CD8^+^T cells [4]. We speculate that glucose restriction will also redirect CD8^+^TILs to use alternative carbon sources, such as lactate acid, fatty acids or ketone bodies, to fuel tricarboxylic (TCA) cycle and oxidative phosphorylation (OXPHOS) for ATP production. Increased energy production through OXPHOS not only robs TILs of the anabolic pathways that feed off intermediates of glycolysis but it also requires oxygen, which can become limiting in solid tumors.

Various studies have shown that aging may pose additional challenges to TA-specific CD8^+^TILs, which could further dampen the efficacy of cancer immunotherapy. First, metabolic fitness declines upon aging. Mitochondrial functions decrease due to accumulating mitochondrial DNA mutations. This in turn limits the aging cells' ability to gain energy through the TCA cycle and OXPHOS. As shown in C. elegans [5], increased reliance on glycolysis in aged cells may be further driven by changes in two key metabolic enzymes, phosphoenolpyruvate carboxy-kinase (PEPCK) declines while pyruvate kinase (PK) reciprocally increases in the aged. PEPCK is an enzyme of cataplerosis, a process that is essential to maintain the balance of the TCA cycle by removing excess amounts of oxaloacetate. Lack of PEPCK thus reduces mitochondrial respiration while increase of PK, which converts phosphoenolpyruvate to pyruvate, promotes energy production through glycolysis. Needless to say that this is not a viable option for TILs that try to exert effector functions within a glucose-deprived TME.

Secondly, the low-grade chronic inflammation in the aged shapes the TME. Cytokines and reactive oxygen species (ROS) secreted by inflammatory cells promote tumorigenesis. Accelerated growth of cancer cells increases competition for glucose and exacerbates hypoxia within the TME; both may further dampen the effector functions of TA-specific CD8^+^TILs. Production of pro-inflammatory cytokines increases in the aged. Cytokines such as IL-6 and TNF-α recruit ISCs such as myeloid-derived suppressor cells (MDSCs), tumor-associated macrophages (TAMs) and regulatory T cells (Tregs) into the TME and promote their activation. MDSCs deplete L-arginine, tryptophan and cysteine through expression of arginase, indoleamine 2,3-dioxygenase (IDO) and cysteine uptake transporter [6]. As these amino acids are essential for T cell activation and proliferation, their decrease within the TME impairs TIL functions and survival. Moreover, both tumor cells and tumor stromal cells have abnormally high rates of glycolysis, which through secretion of lactate creates an acidic TME. This in turn blocks the discharge of lactic acid by activated CD8^+^TILs, which affects both their metabolism and functions [7].

Can we improve the efficacy of cancer immunotherapy in the aged by increasing the metabolic fitness of TILs? Caloric restriction, known to reduce the incidence of cancers in experimental animals, increases mitochondrial performance. Notwithstanding this may not help cancer patients, which commonly already suffer from cachexia/anorexia. Drugs that instruct T cells to reduce glycolysis and increase catabolism of other nutrients that are more abundant within the TME such as fatty acids could provide a better solution and should be explored as facilitators of active cancer immunotherapies in the aged.
